# Transcriptomic response to soybean meal-based diets as the first formulated feed in juvenile yellow perch (*Perca flavescens*)

**DOI:** 10.1038/s41598-020-59691-z

**Published:** 2020-03-04

**Authors:** Megan M. Kemski, Chad A. Rappleye, Konrad Dabrowski, Richard S. Bruno, Macdonald Wick

**Affiliations:** 1 Department of Food Science and Technology, The Ohio State University, Columbus, OH USA; 2 Department of Microbiology, The Ohio State University, Columbus, OH USA; 3 School of Environment and Natural Resources, The Ohio State University, Columbus, OH USA; 4 Department of Human Sciences, The Ohio State University, Columbus, OH USA; 5 Department of Animal Sciences, The Ohio State University, Columbus, OH USA

**Keywords:** RNA sequencing, Ichthyology

## Abstract

With increasing levels of fish meal (FM) protein in aquafeeds being replaced with soybean meal (SBM) protein, understanding the molecular mechanisms involved in response to alternative diets has become a critical concern. Thus, the goal of this study was to examine transcriptional differences in the intestine of juvenile yellow perch through RNA-sequencing (RNA-seq), after their initial introduction to a formulated diet with 75% SBM protein inclusion for 61 days, compared to those fed a traditional FM-based diet. Transcriptomic analysis revealed a concise set of differentially expressed genes in juveniles fed the SBM-based diet, the majority of which were intrinsic to the cholesterol biosynthesis pathway. Analysis of total body lipid and cholesterol levels were also investigated, with no between-treatment differences detected. Results of this study demonstrate that in response to SBM-based diets, yellow perch juveniles up-regulate the cholesterol biosynthesis pathway in order to maintain homeostasis. These findings suggest that the upregulation of the cholesterol biosynthesis pathway may negatively impact fish growth due to its large energy expenditure, and future studies are warranted.

## Introduction

Of the many plant proteins, soybean protein in the form of soybean meal has become a leading fish meal replacement in aquaculture. It has been widely used due to its high protein content, relatively well-balanced amino acid profile and lower cost compared with fish meal^1^. The incorporation of soybean meal in fish diets could alleviate some of the sustainability and cost problems associated with fish meal use^2^. However, while soybean meal is an inexpensive alternative to fishmeal, it contains certain undesirable nutritional characteristics. These include high carbohydrate levels, the presence of isoflavones, low methionine levels and anti-nutritional factors (lectins, oligosaccharides, saponins and trypsin inhibitors) that are reported to impede protein digestion, impair immune responses, and cause intestinal inflammation that hinders fish growth^2–4^. Among fish species, yellow perch are particularly sensitive to the anti-nutritional factors within soybean meal, and growth performance was hindered as a result of inclusion levels within the diet of 40% or higher^5^.

SBM and other plant proteins can be incorporated into adult European seabass (*Dicentrarchus labrax)* diets at levels of 50% and higher without adverse effects on growth, yet there are still limitations, especially for juvenile fish^6^. For example, decreased growth occurs in juvenile Atlantic salmon (*Salmo salar*) and rainbow trout (*Oncorhynchus mykiss*) that have been fed diets with more than 50% fish meal replacement with plant proteins^7,8^, as well as in juvenile rainbow trout that were fed a completely plant-based diet^9^. In agreement, we reported that juvenile yellow perch fed a diet with 75% FM protein replacement with soybean meal protein displayed significantly reduced growth compared with those fed FM-based diets, yet adults fed the same diets had similar growth between the two groups^10^. This suggests that juvenile fish are more susceptible to adverse growth performance, and that further investigation into the molecular mechanisms by which diet composition regulates growth is needed.

A major topic of investigation in the past few years has been to understand fish responses, and the molecular mechanisms involved in response to new diets^11,12^ in hopes of improving their use among sensitive species and during developmental stages. Transcriptomic studies provide tissue-specific, gene transcription patterns of the physiological responses of fish to diets with various FM replacements. There have been several publications examining transcriptional changes after FM is replaced with plant-based proteins in diets of rainbow trout or Atlantic salmon in various tissues; liver^9,13–15^, whole body^16^, brain^17^, and intestine^18–23^. Of the studies that investigated dietary SBM replacement on the transcriptome, Sahlmann *et al*.^23^ looked at initial responses in the distal intestine after feeding Atlantic salmon a diet with 20% SBM on day 1, 2, 3, 5 and 7. The most prominent gene transcription changes occurred after day 3 and 5, in immune response-related transcripts. Fish sampled on day 5 and 7 showed down regulation of transcripts related to metabolic processes, endocytosis, and exocytosis which indicated SBM diets had impaired digestive and metabolic functions. In a longer term study, De Santis *et al*.^15^ fed Atlantic salmon graded levels of SBM (0, 10, 20 and 30%) for 12 weeks. Gene transcription was analyzed in the liver and distal intestine, and results from the intestine showed that of the fish fed the 30% SBM, they had an increase in immune related, anti-inflammatory proteins, and of pathways involving protein synthesis and cell proliferation. Genes that were down regulated were those involved with metabolic pathways (lipid, sterol and vitamin metabolism, digestion and absorption), along with those in pathways associated with phagocytosis. The authors were unsure if down regulation of these processes were caused by tissue damage, or the presence of anti-nutritional factors in SBM that interfere with normal absorption of nutrients and are known to cause hypocholesterolemia^15,24^.

To the best of our knowledge, there are no transcriptional studies in yellow perch concerning dietary ingredients and FM replacements. Additionally, there are a lack of studies addressing transcriptional changes in juvenile fish, which may provide answers as to why such growth differences are seen when compared to adults. Thus, the goal of this study was to examine transcriptional responses in the mid-intestine of juvenile yellow perch after their first introduction to a formulated diet with 75% SBM protein replacement for 61 days compared to those fed a traditional FM-based diet.

## Results

### Feeding trial

**Figure 1:**
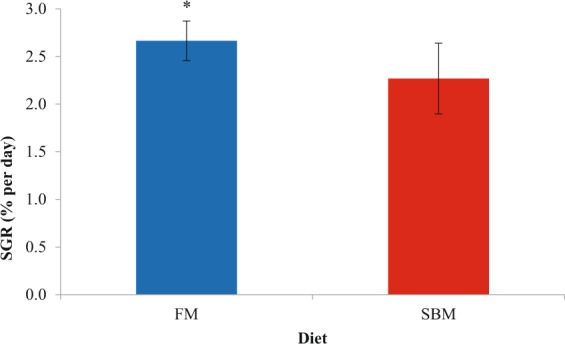
Specific growth rate (SGR) of juvenile yellow perch after being fed either a FM or SBM diet for 61 days. Measurements were taken from all individual fish (n = 50/tank) among 12 total tanks; 6 fed FM and 6 fed SBM-based diets. SGR = (*Ln (weight gain(g))/days) × 100* and represents growth rate in % per day. Data are presented as mean ± standard deviation and was analyzed by a two-way *t*-test. Asterisks denotes significant differences between the two groups (p = 0.035).

Survival and body weight data for juveniles after the 61-day feeding trial are presented in Table 1 and specific growth rate (SGR, Eq. 1) is presented in Fig. 1. At the end of the trial, significantly lower weight gain (p = 0.023, degrees of freedom (DF) = 11.37) and SGR (p = 0.035, DF = 10.43) was seen in the SBM fed group compared to the FM fed group. However, no significant differences were seen between the groups in terms of survival (p = 0.289, DF = 11.95) and weight gain percent (p = 0.106, DF = 11.99).

**Table 1:** Growth performance results of the 61-day feeding trial.

Diet	Starting Weight (g)	61 Days Weight (g)	Weight Gain (g)	Weight gain (%)	Survival (%)
FM	0.13 ± 0.03	5.11 ± 0.63*	4.98 ± 0.62*	3887 ± 838	91 ± 5
SBM	0.13 ± 0.03	4.11 ± 0.81	3.98 ± 0.79	3105 ± 839	84 ± 5

Juvenile yellow perch were fed either a fish meal-based diet (FM) or a soybean meal-based diet (SBM). Measurements were taken from all individual fish (n = 50/tank) among 12 total tanks; 6 fed FM and 6 fed SBM-based diets. Data are the mean values ( ± SD) of individual fish in terms of weight (g), weight gain (g or %), and survival (%). A pooled two-tailed *t*-test was run to compare dietary treatment groups, and values within a column with an asterisks (*) denote significant (p < 0.05) differences between groups.

### Transcriptomic responses

**Figure 2:**
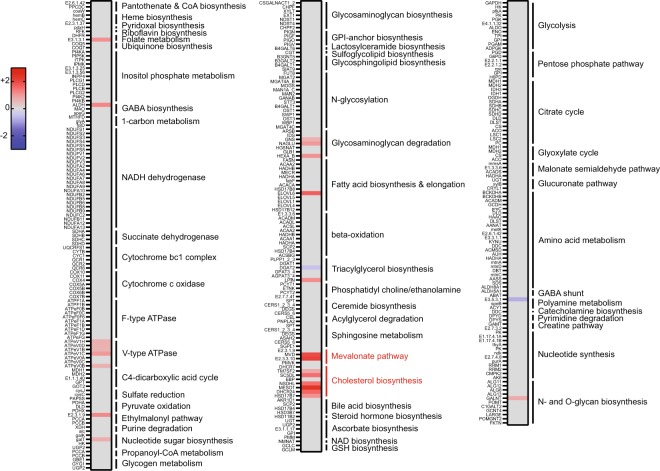
KEGG-module clustering of metabolic genes. Yellow perch orthologs of metabolic genes were mapped to KEGG modules. The relative expression between SBM-fed and FM-fed perch is represented by the heat map (shown as the log_2_(fold change) for SBM compared to FM). Only genes that were significantly different between groups (p < 0.05) are colored.

Transcriptomic analysis was completed and compared among intestinal samples from 6 FM and 6 SBM fed fish. In the absence of a reference genome for yellow perch, transcripts were assembled de novo from the RNA-seq reads. Repetitive sequences were removed along with low confidence transcripts (average FPKM value of <2.0). After filtering for low confidence assemblies, 13,630 total transcripts were assembled from our intestinal transcriptome. Differential expression analysis was performed by mapping reads to the assembled transcripts. All responses of the intestinal transcriptome to the SBM-based diet were expressed relative to the FM-based diet. A total of 1025 gene isoforms were significantly differentially expressed (p < 0.05), using an exact test in the edgeR statistical package following normalization of counts. Of those, 53 transcript isoforms representing 33 genes with differential expression of at least 3-fold change (log_2_ (fold change) of ±1.5) were found (Supplemental Data File S1 -Provides all transcript isoforms and FPKM values after initial filtering along with subsequent filtering steps). As our focus was on nutritional aspects of SBM-based diets, we annotated transcripts by their KEGG orthology and subsequently mapped the metabolism-related genes to KEGG modules to identify metabolic clusters with differentially expressed genes (Fig. 2). Using this metabolic analysis, multiple genes within the cholesterol and mevalonate biosynthesis pathways were upregulated in tissues from fish fed SBM. Genes encoding components of the V-type ATPase were also upregulated, but none more than 2-fold despite the statistical significance, questioning the biological relevance of the gene expression changes in this module. However, as the mevalonate pathway produces precursors for the biosynthesis of sterols, these results strongly implicated changes in cholesterol homeostasis as a consequence of the SBM-based diet.

### Up-regulation of genes found in SBM fed fish

**Figure 3:**
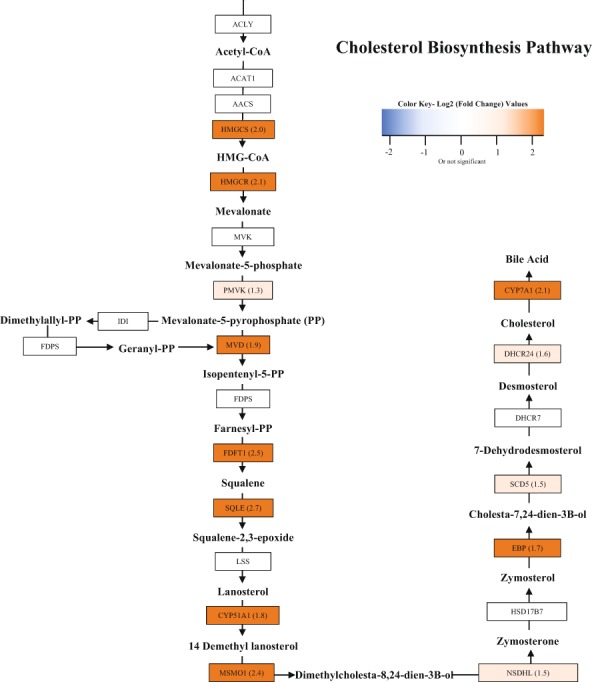
Cholesterol biosynthesis pathway and gene expression identified through RNA-seq. Major metabolic intermediates of the cholesterol biosynthesis pathway are presented along with reaction facilitating enzymes in boxes. RNA-seq results of the Log_2_(fold change) for SBM compared to FM for enzymes that were found to be significantly different are provided. Color scale bar describes the various colors presented in the pathway.

There were 42 isoforms representing 27 genes up-regulated at least 3-fold in juveniles fed a SBM-based diet as their initial feed. Nine of these play integral roles in the cholesterol synthesis pathway (Table 2), consistent with the KEGG module analysis (Fig. 2). Two of these genes are known to be rate limiting in both the cholesterol biosynthesis pathway (i.e. 3-hydroxy 3-methylglutaryl-CoA reductase, HMGCR) and in bile acid production (i.e. cholesterol 7-alpha -monooxygenase, CYP7A1). With the identification of cholesterol biosynthesis by two analyses, we examined the transcriptional changes of all the genes encoding enzymes involved in cholesterol biosynthesis and present them in Fig. 3. The SBM-based diet also stimulated genes involved in lipid metabolism and transport, which also contribute to cholesterol transport and regulation of circulating levels, such as long chain fatty acid elongase and proprotein convertase subtilisin/kexin-type 9 (Table 2). Other genes upregulated in SBM fed juveniles included various genes involved in cellular activity and immune defense (Table 2). However, these non-cholesterol/lipid-related genes did not cluster into obvious pathways, and thus the implications of these other transcriptional changes are uncertain.

**Table 2:** RNA-seq results of the top differentially expressed genes in the mid intestine between fish fed the SBM-based diet and FM-based diet.

Up-Regulated Genes	Gene Symbol	Log_2_(FC)	P-value
**Cholesterol Biosynthetic Pathway**			
squalene monooxygenase	SQLE	2.67	0.004
farnesyl-diphosphate farnesyltransferase 1	FDFT1	2.53	0.005
methylsterol monooxygenase 1	MSMO1	2.42	0.002
cholesterol 7-alpha-monooxygenase	CYP7A1	2.05	0.008
3-hydroxy-3-methylglutaryl-coA synthase 1	HMGCS1	2.03	0.024
mevalonate diphosphate decarboxylase	MVD	1.89	0.012
hydroxymethylglutaryl-CoA reductase	HMGCR	2.14	0.004
lanosterol 14-alpha demethylase	CYP51A1	1.82	0.005
emopamil-binding protein	EBP	1.69	0.002
**Lipid Transport and Metabolism**			
polyunsaturated fatty acid elongase	ELVOL5	1.98	0.045
long chain fatty acid elongase 6	ELVOL6	1.69	0.023
low-density lipoprotein receptor-related protein 2-like	LRP2	1.78	0.012
phthiocerol synthesis polyketide synthase type I	PPSD	1.71	0.044
proprotein convertase subtillisin/kexin type 9	PCKS9	1.60	0.001
**Other Metabolic Processes**			
cathepsin B	CTSB	1.91	0.001
transcobalamin 2-like	TCO2	1.74	0.002
renin binding protein	RENBP	1.71	0.006
natterin 3-like	NATT3	1.53	0.004
perilipin-3-like	PLIN3	1.52	0.021
**Cellular Processes**			
dihydropyrimidinase related protein 5-like	DPY5	2.66	0.017
Eukaryotic translation initiation factor	EIF5	1.51	0.002
LaminA	LMNA	2.63	0.002
**Immune Response**			
ubiquitin carboxyl-terminal hydrolase 10-like	USP10	2.98	0.004
carcinoembryonic antigen related cell adhesion molecule 1-like	CAECAM1	2.51	0.001
butyrophilin subfamily 2 member A2	BTN2A2	2.29	0.002
carcinoembryonic antigen related cell adhesion molecule 5-like	CAECAM5	1.68	0.017
NACHT, LRR and PYD domains containing protein 12 like	NLRP12	1.57	0.008
**Down-Regulated Genes**	**Gene Symbol**	**Log**_**2**_**(FC)**	**P-value**
**Lipid/Cholesterol Regulation**			
mid-1 interacting protein 1-B like	M1IP1	−2.16	0.017
bile salt export pump-like	BSEP	−1.51	0.007
**Cell Signaling**			
high choriolytic enzyme 1-like	HCEA	−3.51	0.022
C1q tumor necrosis factor related protein 3-like	C1QTNF3	−2.69	0.019
cytochrome P450 1A	CYP1A	−2.06	0.031
neurexophilin 2	NXPH2	−1.80	0.015
peroxisomal 2,4 -dienoyl CoA Reductase -like	PECR	−1.65	0.029

All genes were found to be significantly different between treatments (p < 0.05), and expression was consistent among replicates within a treatment.

### Down-regulation of genes found in SBM fed fish

Only 8 isoforms were identified as down-regulated in fish fed SBM-based diets. These represent 6 down-regulated genes which were involved in cell signaling, such as neurexophilin 2 (NXPH2) and C1Q tumor necrosis factor related protein C1QTNF (Table 2). Additionally, some lipid biosynthesis genes, such as mid-1 interacting protein 1-B (M1IP1) that regulates the lipid biosynthesis pathway, and bile salt export pump (BSEP) which is the major transporter for the secretion of bile acids from hepatocytes into bile, were all found to be down-regulated in SBM fed juveniles (Table 2).

### Quantitative real-time PCR validation

**Figure 4:**
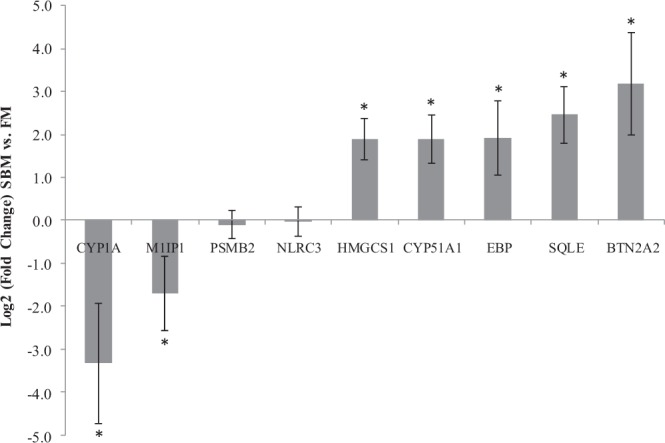
Quantitative real-time PCR confirmation of gene expression in the mid intestine of fish fed SBM-based diets compared to fish fed FM-based diets for 61 days. Data are presented as the mean Log_2_(Fold Change) ± standard deviation of twelve fish, and asterisks (*) indicate significant differences between treatments (p < 0.05), determined by a two-way *t*-test.

To validate the transcriptome analysis, quantitative real-time PCR (qRT-PCR) was performed on 11 genes (Table 3), both up and down regulated using RNAs extracted from the mid intestine of fish fed SBM-based diets compared to those fed FM-based diets for 61 days. Genes of various expression levels were randomly selected from the top differentially expressed genes determined through RNA-seq. Additionally, genes were selected if there was sufficient sequence information provided for the gene of interest, since the genome for yellow perch was not available at the time of analysis. Housekeeping genes were chosen based on those described in previous literature^25–27^. Expression levels were normalized to RNA-Polymerase (RNApol), as this was deemed to be the most stable gene based on both RT-qPCR results as well as RNA-seq results. Results from the qRT-PCR analyses are presented in Fig. 4 and were found to be consistent with the expression trends observed in the RNA-seq data.

**Table 3:** Primers used for Real-Time qPCR expression analysis. Forward and reverse sequences are given, along with product size in base pairs (bp) and annealing temperature (Tm°C).

Gene Name	Forward	Reverse	Product Size (bp)	Tm (°C)
**Down-Regulated**				
CYP1A	GACAGGCCAGTGGAGATGG	TTTGTGCAGAGGATCGTGG	200	58
M1IP1	CTGATGAGGTGTGGGACTGC	GGCCTTGTGGTCATCTTGGA	128	60
PSMB2	ATGGCCCCGGTCTCTACTAC	ACACGCATGAGGCATTTCTCT	146	60
NLRC3	AAATGGTCTGACTGGTCGCT	CATCAGAGCAGAACAGACTCCA	197	59
**Up-Regulated**				
HMGCS1	GATTGGAGATCAGCAGGGAGG	CCAAGGTGGCAAAAGTACAACTC	182	60
CYP51A1	TGGGGCGTGTGTTTTGAGAG	CACCACGGATGTCAGGTTGT	129	55
EBP	CTCCACGGTCCCAACAAGAG	TGGTGTCCGTGCTATCTCTG	188	57
SQLE	AGCCCCGTCGTCATAGAGAT	GGCGATGCGTACAACATGAG	120	55
BTN2A2	AGCCTGCTGTTCATCCTCAT	TGATCCAGAAGAAGCCGAGG	187	55
**Housekeeping**				
18S	TACAGTGAAACTGCGAATGG	GCATGGGTTTTGGGTCTG	153	60
ACTB	GGCCAACAGGGAAAAGATGA	ACCGGAGTCCATGACGATAC	130	59
EF1α	TGACAACGTCGGCTTCAACA	GGGTGGTTCAGGATGATGAC	135	60
RNAPOL	GCCATGACACCCAGCTAACA	GCAACGTGTGTCCGTGTTTT	157	60
RPS20	AGCCGCAACGTCAAGTCT	GTCTTGGTGGGCATACGG	98	60

### Whole body lipids and cholesterol analysis

**Figure 5:**
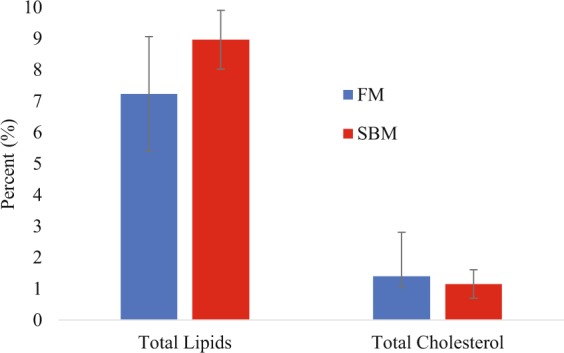
Whole body concentration (% wet weight) of total lipid and cholesterol levels in fish fed FM (n = 3) and SBM diets (n = 3). No significant differences were found between groups, determined by a two-way *t*-test.

To determine if transcriptional responses caused physiological changes due to SBM replacement of FM in the juvenile diets, lipid and cholesterol analysis was conducted on the two populations of fish. Whole body lipid analysis revealed that the FM fed fish had a total lipid content of 8.97 ± 0.94%, whereas SBM fed fish had a total lipid content of 7.23 ± 1.83%, which were not significantly different (p = 0.219, DF = 4) (Fig. 5). Additionally, total body cholesterol content of juveniles fed the SBM-based diet was 1.40 ± 0.36%, compared to 1.15 ± 0.45% in fish fed the FM based diet. Again, no significant differences were found between groups (p = 0.495, DF = 4) (Fig. 5).

## Discussion

As growth of the aquaculture industry continues, the need for a cost effective, sustainable fish meal replacement is imperative. By having a better understanding of the molecular mechanisms and gene expression changes that occur when plant-based proteins are introduced into piscivorous fish diets, it allows researchers to create optimal feeds better suited for these fish. There have been numerous studies that examined the transcriptional response of fish to partial FM replacements with plant-proteins^17,19,22^, SBM^15,28^, various mixtures of SBM and other plant based protein sources^20^, and even complete replacement of fish meal/fish oil with plant sources^9,29^. However, the majority of the aforementioned studies analyzed transcriptional changes within the liver or distal intestine, and have also reviewed by Martin *et al*.^11^.

Results of the current study’s feeding trial showed that after 61 days, juveniles fed the SBM diet had lower average final weight when compared to fish fed the FM diet. It is important to note that feeding rates among all tanks were monitored and controlled so that both diet groups were given the same amount of food daily, and all food was consumed. Additionally, while fish weight is a convenient measurement of growth, fish growth is a combination of many factors. For example, different tissue composition (e.g., fat versus muscle) could result in different sized fish but with similar weights. Therefore, it is worth noting that some of SBM-fed fish had extensive visceral fat surrounding the intestinal tract causing distended abdomens that were not observed in FM-fed fish. Growth results from the present study, however, are consistent with other studies in which fish meal proteins were replaced at high levels with plant based proteins, and fed to juvenile Coho salmon (*Oncorhynchus kisutch*)^30^, Atlantic salmon and rainbow trout^7–9^.

Conversely, in terms of overall weight gain percent in the current study, fish fed the SBM-based diet were not significantly different from the FM fed fish. These results are promising because weight gain of the SBM fed juveniles has significantly improved when compared to juveniles fed the same SBM diet over an equal duration in our previous study^10^. Although, juveniles in the current study are progenies from broodstock fish from Kemski *et al*.^10^ that had been nutritionally programmed over 3 growing seasons, which we hypothesize enhances acceptability and utilization of soybean-meal based diets. Additionally, stocking weight at the time of transition to formulated feed is important, especially in yellow perch, thus stocking juveniles at 0.125 g in the current study, we believe, was crucial to diet acceptance.

The intestine plays a vital role in digestion and absorption of nutrients and thus is highly sensitive to dietary modifications, especially in carnivorous fish. In a study by Tacchi *et al*.^22^, the authors examined transcriptional changes in the mid intestine, liver and skeletal muscle of Atlantic salmon after being fed a plant-based diet, and found the greatest transcriptomic response in the intestine of the three tissues studied, reflecting its sensitivity to dietary changes. The intestine is divided into three discrete regions; the proximal region that also includes the pyloric caeca, the mid and distal intestine. While nutrient absorption occurs in all regions, it is highest in the proximal/mid region^31^. Previous studies have shown that the distal intestine is the main region for enteritis due to SBM inclusion in the diet^3,28,32,33^. Therefore, the focus of this experiment was on transcriptional changes in response to a SBM-based diet in the mid intestine, so that metabolic changes could be highlighted rather than enteritis.

The majority of genes found up-regulated in the mid intestine of juveniles fed the SBM diet were involved in cholesterol biosynthesis (Figs. 2 and 3). A strength of our findings is that transcriptional changes were limited to only a few of the thousands of genes expressed by intestinal tissue. Due to the stringency we applied to determine statistically valid expression changes, we identified 9 of the main enzymes in the cholesterol pathway, some being rate limiting. This also reduces a lot of noise that is usually seen with less stringent fold-change cutoffs. In addition, the finding that several genes within the same pathway are upregulated significantly corroborates the conclusion that the transcriptional response is specific.

It is well known that cholesterol is only found in animal products, and that replacements with plant proteins will have reduced dietary levels of cholesterol, even if this is not indicative of circulating cholesterol. However, with higher inclusion levels of plant proteins, naturally occurring plant phytosterols increase. Research has also shown that cholesterol intake in the intestinal lumen may even be impaired by the presence of phytosterols in SBM^3,24,34^, and hypocholesterolemia can occur^35^. Fish are able to synthesize cholesterol de novo from acetate, yet the potential for synthesis can vary and is not well defined^36^. Cholesterol homeostasis is regulated by cholesterol intake and modified by the de novo biosynthesis pathway as well as conversion and excretion of bile acids. When intake of cholesterol is low, biosynthesis increases to maintain sterol levels. Biosynthesis is regulated by 3-hydroxy- 3-methylglutaryl reductase (HMGR), a rate-controlling enzyme in the mevalonate pathway^37^. Another gene that maintains cholesterol homeostasis is cytochrome P4507A1 (CYP7A1). Its activity is regulated by the flux of cholesterol through the liver, and when cholesterol levels are high, its activation allows for the conversion of cholesterol to bile acids^38^. Consequently, circulating cholesterol levels in the body and bile acid metabolism are closely linked. Our results, which showed no difference in total body cholesterol between treatments, suggest that yellow perch increase cholesterol biosynthesis to maintain homeostasis in response to the low dietary cholesterol levels in the SBM diets. Thus, an explanation to CYP71A being upregulated in the SBM-fed fish along with other genes found in the cholesterol biosynthesis pathway is that they are producing enough cholesterol and when circulating levels get high, this gene is also upregulated to help maintain homeostasis. This is in agreement with other studies in which HMGCR and CYP7A1 were up-regulated in response to non-FM based diets in Atlantic salmon^35^, rainbow trout^16,29^, European seabass^39^ and yellowtail^40^.

It has been hypothesized that reduced cholesterol levels can cause a reduction in growth performance in fish, and studies have reported significantly improved growth when cholesterol was supplemented in diets that contained high levels of SBM in catfish^41^; turbot^42,43^; and rainbow trout^36^. Norambuena *et al*.^44^. along with Turchini *et al*.^45^. discuss the fact that the cholesterol biosynthesis pathway is a high energy expenditure with a significant metabolic cost. One cholesterol molecule is composed of 18 Acetyl-CoA, 18 ATP, 16 NADPH, and 4 O_2_ molecules. These compounds are also highly involved in fatty acid metabolism, which contributes to muscle growth as well as the citric acid cycle. Thus, an increase in the biosynthesis pathway is redirecting the major metabolic compounds from these cycles and using them for cholesterol production. This may be one of the reasons that hindered fish growth is seen in fish when fed plant-based diets, and further studies on this topic are warranted.

Genes involved in lipid metabolism and transport were also up regulated in fish fed the SBM diet, which are consistent with previous studies^27,29,46^. Lipid and cholesterol transport are closely related and specific genes that were found up-regulated, such as proprotein convertase subtilisin/kexin-type (PCSK9) and low-density lipoprotein receptor-related protein 2-like (LRP2) are involved in LDL binding as well as cholesterol homeostasis. This transcriptional up-regulation of genes involved in lipid transport/metabolism in connection with cholesterol homeostasis was also seen in studies by Krol *et al*.^20^ and Tacchi *et al*.^22^ after Atlantic salmon had been fed plant-based diets. These results suggest that regulatory mechanisms exist in the intestine, as well as the liver of fish fed plant-based diets in order for them to compensate for reduced dietary cholesterol levels and lipid export rate^27^.

Despite changes in sterol/lipid biosynthesis gene expression, overall total body lipid content of these fish was not significantly different between the SBM and FM fed juveniles after 61 days of feeding. Additionally, whole body cholesterol content was not significantly different between SBM and FM fed fish. Together these results indicate that increased cholesterol biosynthesis compensates for low dietary levels. Conversely, total body cholesterol results found in the current study differ from those found in studies by Deng *et al*.^36^ and Zhu *et al*.^47^, in which the authors measured cholesterol levels in rainbow trout after being fed diets with varying amounts of added cholesterol or complete FM replacement with plant proteins, respectively. The authors found that cholesterol content was significantly reduced in rainbow trout fed a diet that was completely marine-free^47^, or diets with less than 1.2% cholesterol^36^. Furthermore, there have been numerous studies in which plasma cholesterol content was significantly reduced after FM was replaced with plant proteins in rainbow trout^32,47^, European seabass^48^, and Atlantic salmon^24^. This occurrence might be explained by the fact that excess cholesterol in the body is stored as cholesterol esters in cytoplasmic lipid droplets^49^. Because of the reduced cholesterol content in the plant based diets, levels in the plasma remain low, causing an increase in cholesterol biosynthesis, thus resulting in cholesterol accumulation through lipid droplets^27^.

## Conclusions

Results from the present study reveal that juvenile fish fed SBM-based diets up-regulate genes involved in the cholesterol biosynthesis pathway and fatty acid transport in the mid intestinal region, compared to fish fed FM-based diets. Nonetheless, total body cholesterol levels in the SBM-fed fish were not significantly different than the FM-fed fish, suggesting that fish on SBM-based diets increase de novo cholesterol biosynthesis to compensate for dietary deficiency. The upregulation of the cholesterol biosynthesis pathway may be a partial cause of the reduced weight gain seen in these fish due to the high metabolic requirements for cholesterol synthesis. To the best of our knowledge, this is one of the first studies of its kind to examine the transcriptional response by RNA-seq in the mid intestine of juvenile yellow perch after being fed a SBM-based diet. This demonstrates the capacity of yellow perch to adapt within the first 61 days of introduction to a plant-based formulated feed in order to maintain growth performance.

## Methods

### Yellow perch feeding trial and sampling

Animal care was regulated by the Institutional Animal Care and Use Committee (IACUC approval 2008A0221-R2), and all experiments were performed in accordance with relevant guidelines and regulations. Any handing of fish was performed after sedation by tricaine methanesulfonate anesthesia (MS-222, Western Chemical, Inc. Ferndale, MA), and all efforts were made to minimize suffering. Spawning of broodstock yellow perch occurred in spring 2017, and spawning methods along with embryonic incubation were performed as described in Kemski *et al*.^10^. While the aforementioned paper utilizes broodstock diet history as a breeding scheme, the current study did not evaluate parental diet history in juveniles used for the feeding trial; and fish were evaluated solely on initial formulated diet. After hatching, larval yellow perch were reared for 45–60 days on live feeds (rotifers followed by *artemia* nauplii) until reaching a stocking weight of 0.125 ± 0.023 g, at which time they were stocked among 12, conical tanks (40 L), with 50 fish per tank. Juveniles were allowed to acclimate for 1 week while still feeding on live *artemia*. After the acclimation period, experimental diets were introduced, and fish had an average body weight of 0.133 ± 0.028 g. Two diets were used: 1) control diet with fish meal as the major protein source (FM), 2) soybean meal (3011; Schillenger Genetics Inc.), providing 75% replacement of fish meal protein (SBM)^10,50,51^. Diets were formulated to be isonitrogenous and isoenergetic and were produced using commercial manufacturing technology at the U.S. Fish &Wildlife Service Bozeman Fish Technology Center (Bozeman, MT; Table 4). Six tanks were fed the FM-based diet and six tanks fed the SBM-based diet for 60 days from mid-July to September. Temperatures during this time ranged from 21.9–25 °C and fish were fed three times daily (9:00, 13:00 and 17:00). Diets were given starting at a rate of 7% tank biomass, and re-adjusted daily, equally between tanks, based on tank with the lowest feed intake (feeding rates ranged from 5–7% tank biomass). Each tank’s biomass was collected after 30 days in order to correct and update the feeding rate. Between weighing periods, biomass increase was estimated daily, assuming a feed conversion ratio (FCR) of 1^52^. At the end of the feeding trial, survival, specific growth rate (SGR) and percent weight gain for each diet were determined. Equations are defined below:

1 $${\rm{SGR}}=(\mathrm{Ln}\,({\rm{weight}}\,{\rm{gain}}({\rm{g}}))/{\rm{days}})\times 100)$$

2 $${\rm{Percent}}\,{\rm{weight}}\,{\rm{gain}}={\rm{final}}\,{\rm{weight}}\,({\rm{g}})-{\rm{initial}}\,{\rm{weight}}({\rm{g}})/{\rm{initial}}\,{\rm{weight}}({\rm{g}})\times 100)$$

**Table 4:** Formulation and composition of fish meal (FM) and soybean meal (SBM) diets per 100 g. Diet composition is presented in percent dry matter (%DM).

Ingredient	FM (100 g)	SBM (100 g)
Menhaden Fish Meal^1^	54.09	12.00
Soybean Meal (3011)^2^	—	52.86
Wheat Flour	30.15	14.96
Fish Oil	4.34	8.00
Soybean oil	1.98	0.24
Lecithin	3.00	3.00
CPSP^3^	4.00	4.00
Vitamin mix^4^	1.00	1.00
Mineral mix^5^	0.10	0.10
Vitamin C	0.10	0.10
Choline Chloride	0.06	0.06
Calcium Phosphate	—	1.00
Lysine	—	1.50
Methionine	0.33	0.33
Arginine	0.65	0.65
Threonine	0.20	0.20
**TOTAL**	**100**.**00**	**100**.**00**
**Composition**	**FM (% DM)**	**SBM (% DM)**
Dry Matter	91.7	94.35
Crude Protein	42.4	43.9
Crude Lipid	15.6	13.6
Ash	10.7	6.7
Cholesterol	1.59	0.49

^1^Menhaden special select fish meal, 6% fat, 59.03% protein.

^2^Defatted soybean meal (Schillinger Genetics, West Des Moines, IA.) 1.7% fat, 58.9% Protein.

^3^Soluble fish protein hydrolysate (Sopropeche, Boulogne Sur Mer, France).

^4^Mineral Mix: Five milligrams Se in the form of sodium selenite per kg Bern-hart Tomarelli salt mixture (ICN Pharmaceuticals, Costa Mesa, CA, USA).

^5^Vitamin Mix: Roche Performance Premix composition (g kg-1 of vitamin mixture): vitamin A acetate, 7.56;

cholecalciferol, 0.0055; a- tocopheryl acetate, 66.1; vitamin B12, 0.0013; riboflavin, 13.2; niacin, 61.7; D- pantothenic acid, 22.1; menadione, 1.32; folic acid, 1.76; pyridoxine, 4.42; thiamin, 7.95; J-biotin, 0.31 (Hoffman-La Roche, Nutley, NJ, USA).

On the 61^st^ day, fish were fed twice (8:00 and 11:00) to apparent satiation and sampled 3 hours following the final meal to ensure that the intestinal tract was filled. At the time of sampling, fish were euthanized by an overdose of MS-222 (250 mg/L), weighed and tissues were excised and snap-frozen in liquid nitrogen to be stored at −80 °C for RNA extraction or proximate composition/cholesterol analysis. Twelve fish, one from each tank, were sampled for proximate composition (6 FM fed juveniles and 6 SBM fed juveniles). For RNA analysis, the intestinal tract was removed and a 5 mm portion of the mid-intestine from each individual was gently flushed with 5% saline to remove intestinal contents. A total of 12 fish were sampled for RNA (1 from each tank); 6 FM fed juveniles and 6 SBM fed juveniles.

### RNA sequencing

Total RNA was isolated from each sample (50 mg) using an animal tissue RNA purification kit (Norgen Biotek Corp.,Thorold, ON, Canada) according to the manufacturer’s protocol. Briefly, tissue was homogenized in the provided buffer, treated with Proteinase K and purified on columns. Nucleotide concentrations in each total RNA prep were quantified using an Epoch Microplate Spectrophotometer (BioTek, Winooski, VT) by measuring the absorbance at 230, 260 and 280 nm and purity assessed by the 260:280 ratio. 2 μl RNA was also electrophoresed on a 2% agarose TAE gel in order to determine the integrity of the 18 and 28 S bands and if DNA contamination was present. Half of each RNA sample (~33 ug of RNA) was sent on dry ice to Admera Health, LLC (South Plainfield, NJ) for transcriptomic processing (described below), and the remainder stored at −80 °C until qPCR analysis. RNA integrity was determined using a bioanalyzer prior to library preparation. All samples had a RNA integrity number (RIN) of 8.7 and above.

### Library preparation and de novo transcriptome assembly

Library preparation, mRNA sequencing/annotation and gene expression analysis was completed by Admera Health, LLC (South Plainfield, NJ). For the creation of cDNA libraries, polyA transcripts were captured using paramagnetic beads coupled with oligo d(T) based on the NEBNext® Poly(A) mRNA Magnetic Isolation Module protocol. For first strand synthesis, samples were randomly primed (5’ d(N6) 3’ [N = A, C, G, T]) and fragmented based on manufacturer’s recommendations (NEBext®Ultra™ RNA Library Prep Kit for Illumina®). First strand synthesis was completed using the Protoscript II Reverse Transcriptase with a 40 min extension period at 42 °C. All remaining steps for library construction were used according to the NEBext®Ultra™ RNA Library Prep Kit for Illumina®. Illumina 8-nt dual-indices were used for multiplexing of libraries. Samples were pooled and 150 base paired-end (PE) reads were sequenced, with approximately 68 million passing filter reads per sample (Illumina HiSeq. 3000/4000).

Prior to transcriptome assembly, poor quality reads were removed (Phred Score <30). Transcripts were assembled *de novo* without the use of a reference genome using the Trinity assembler^53,54^. Additionally, a k-mer, or nucleotide sequence dictionary, was constructed with all reads having a k-mer length of 25. Low-complexity and singleton k-mers, along with potential error-prone k-mers were removed. Within the k-mer dictionary, the most frequent k-mer was used as a seed for the first contig assembly, where it was extended in each direction by finding the next closest k-mer with a k-1 overlap. The extension procedure was repeated in both directions until it could not be taken further and the longest linear contig was reported as one transcript. These steps were repeated, continuing with the next most abundant k-mer, until the k-mer dictionary was exhausted, and all possible contiguous sequences were assembled.

### Differential expression analysis

Initial expression analysis was performed by mapping RNA reads to the de novo assembled transcriptome using the Tuxedo suite workflow^55^, utilizing the Tophat algorithm. Repetitive sequences were removed along with low confidence transcript (average FPKM value of < 2.0)^56^. Differential expression was evaluated using the edgeR package in R and the total read counts mapping to each transcript. Reads were normalized by the total number of reads in each sample (calcNormFactors) yielding the counts per million reads (cpm) for each gene. Experimental groups were defined as (1) RNA samples from fish that had been fed FM and (2) RNA samples from the SBM fed fish. The KEGG Automatic Annotation Server (KAAS; https://genome.jp/tools/kaas) was used for the annotation of metabolic genes in the transcriptome. Orthologs were mapped to KEGG modules to identify clusters of differentially expressed genes within single pathways. For KEGG-based annotation, perch transcripts were blasted against various fish species (*Danio rerio*, *Cyprinus carpio*, *Ictalurus punctatus*, *Larimichthys crocea*, *Oreochromis niloticus*, *Oryzias latipes*, *Lates calcarifer*, *Seriola dumerili*, *Salmo salar*, *Salvelinus alpinus*, *Esox lucius*) since yellow perch data was not available. Significance between groups was determined using an exact test after estimation of common and tagwise dispersion. Genes found to be significantly different (p < 0.05) were then considered up- or down-regulated in SBM-fed fish compared to the FM-fed fish if the difference was at least 3-fold (or Log_2_(Fold Change) of at least 1.5). Transcripts that met these two conditions were annotated manually by BLAST search against the NCBI reference nucleotide database (https://blast.ncbi.nlm.nih.gov).

### Quantitative real-time PCR validation

Primers for real-time quantitative PCR (RT-qPCR) were designed using the NCBI Primer-BLAST tool, with gene sequences taken from the annotated transcriptome. Genes of interest and primers used to measure expression are given in Table 3 and included select “housekeeping” genes that showed consistent expression differences between experimental groups and other genes based on previous literature^25–27^ (Table 3).

Total RNA was reverse transcribed using iScript Reverse Transcription Supermix for qPCR (Bio-Rad, Hercules, CA) according to the manufacturer’s protocol. Briefly, 200 ng total RNA was combined with the iScript Supermix and incubated in a thermocycler using the following protocol; 5 minutes at 25 °C, 20 minutes at 46 °C for reverse transcription, and 1 minute at 95 °C. Endpoint PCR was performed to ensure single amplicons were produced of the predicted size. In addition, PCR products were digested with restriction enzymes (restriction fragment length polymorphism (RFLP)) to ensure amplification of the correct sequence predicted from the assembled transcriptome gene sequences. cDNA templates were used at a 1:25 concentration in a standard 50 μl PCR master mix with Platinum Taq (Thermo Scientific, Waltham, MA) and 10 mM of each gene-specific primer. After an initial hold at 95 °C for 3 ½ minutes, reactions were cycled 35 times at 55 °C for 30 seconds, and 72 °C for 1 minute in a Biorad T100 thermal cycler (Biorad, Hercules, CA). For RFLP, 10 μl of the PCR products were incubated overnight at 37 °C with NEB restriction enzymes (New England Biolabs Inc., Ipswich, MA) and compatible buffer, and visualized by agarose gel electrophoresis. Each PCR product was cut with 3 different restriction enzymes, to ensure that the predicted sequence matched the amplicon generated.

Quantitative PCR was performed with a CFX382 Touch™ Real Time PCR Detection System (Bio-Rad, Hercules, CA). Each 10 μl reaction mixture contained 2 μl cDNA at a 1:10 dilution, 2 μl PCR-grade water, 0.5 μl each primer (500 nM final concentration), and 5 μl SsoAdvanced ™ Universal SYBR® Green Supermix (Bio-Rad, Hercules, CA). The following program was used for amplification; 95 °C for 2 minutes, followed by 39 cycles of 95 °C for 10 sec, 60 °C for 30 sec, 72 °C for 30 sec and a final extension time at 72 °C for 5 minutes. Melt curves for each PCR product were analyzed following the run to ensure that only a single product had been amplified. PCR efficiency was validated using 10-fold dilutions of cDNA. Housekeeping genes were evaluated by the overall co-variance (CV) of each gene as previously described^18,57,58^, along with the geNorm of each sample according to Vandesompele *et al*.^59^. These methods measured the stability of each reference gene by calculating pair-wise variations among standard error or among other reference genes^60^.

### Lipid and cholesterol analysis

Whole body fish samples underwent total lipid extraction according to the Folch^61^ method. Two fish per dietary treatment group were combined, pulverized under liquid nitrogen, and 1 g was used for lipid extraction. Three replicates were done per treatment group (SBM n = 3 and FM n = 3) for this analysis, and results are presented in % wet weight. The lipid extractions were then resolubilized to determine total cholesterol content using the Cholesterol Reagent Kit (Pointe Scientific, Canton, MI), with minor modifications. Briefly, 500 μl of chloroform/Triton X-100 mixture (100:1 v/v) was used to solubilize lipid extracts, which were then dried again under nitrogen gas, and subsequently dissolved in 250 μl water while shaking in a water bath for 15 min (37 °C). Subsequent steps were carried out according to manufacturer’s instructions, and absorbance was read in a Synergy H1 Hybrid Multi-Mode spectrophotometer (BioTek, Winooski, VT) at 37 °C and 500 nm.

### Statistical analysis

Results are expressed as mean ± standard deviation (SD). Individual tanks represented the statistical unit, and for fish growth, transcriptomic and q-PCR analysis replicates of each group were measured as follows, FM; n = 6, and SBM; n = 6. Lipid and cholesterol analysis combined two fish from the same tank, and replicates were taken from three different tanks per dietary treatment, thus, FM; n = 3 and SBM; n = 3. All data were checked for normality by the normal goodness of fit test, and groups were compared by a pooled, two-tailed *t* test (JMP Pro v14, SAS Institute, Cary, NC). RNA-seq data was analyzed in edgeR, as previously mentioned, by using an exact test after estimation of common and tagwise dispersion. Survival of fish during the feeding trial was arcsine transformed for normality. Data was considered significant when p < 0.05.

## Supplementary information

Dataset 1.
